# Neglected Incisional Hernia Becomes a Life-Threatening Emergency: A Case Report

**DOI:** 10.7759/cureus.82993

**Published:** 2025-04-25

**Authors:** Saurabh Kumar, Sakshi Jaiswal, Sanjay Kumar Saroj, Satyanam Kumar Bhartiya

**Affiliations:** 1 General Surgery, Institute of Medical Sciences, Banaras Hindu University, Varanasi, IND

**Keywords:** acute intestinal obstruction, bowel evisceration, emergency laparotomy, incisional hernia, spontaneous rupture

## Abstract

Although complications are common following abdominal surgeries, the nontraumatic spontaneous rupture and obstruction with skin gangrene of an incisional hernia leading to bowel evisceration is unusual. This report describes the case of a 40-year-old female with a long-standing incisional hernia that presented as a spontaneous evisceration of an obstructed bowel with evidence of skin rupture and gangrene. She was managed with an emergency laparotomy with myofascial anterior component separation and onlay mesh repair.

## Introduction

Most incisional hernias require surgical treatment. If left untreated, these hernias pose various obstructive and non-obstructive complications, including serious complications like bowel incarceration, obstruction, gangrene, and even perforation [1]. It is important to note that in low-resource countries, incisional hernias are often neglected for long periods of time [2]. However, few cases have been reported where an incisional hernia presented as a spontaneous bowel evisceration [1]. This report describes a unique case of a long-standing incisional hernia rupture in a 40-year-old woman who presented with signs of acute intestinal obstruction.

## Case presentation

A 40-year-old multiparous woman with obesity presented to the emergency room (ER) with spontaneous evisceration of the bowel that had lasted for 12 hours. It was also associated with multiple episodes of vomiting; this was non-projectile, bilious, and contained food particles with obstipation.

The patient had developed an incisional hernia following an exploratory laparotomy for perforation peritonitis eight years ago. Following the operation, she developed a surgical site infection and progressed to complete fascial dehiscence. The wound was managed with serial dressing and healed by secondary intention. Soon after, she noticed a globular swelling over the middle scar, but she ignored it and did not seek any further treatment.

Twenty-four hours before arriving at the ER, she felt a sensation of “giveaway” in her abdomen after lifting a heavy bucket. She noticed a small amount of clear discharge from the lower part of her abdominal swelling. Twelve hours after the initial 'giveaway', she had a bout of coughing, after which she felt another ‘giveaway’, and then she saw that a large (huge/big) segment of bowel had protruded from her abdomen. This alarmed her, and she sought emergency medical attention in a district hospital. After initial resuscitation, the hospital referred the patient to a higher centre for definitive management. In the meantime, she had already developed vomiting and absolute constipation, which indicated a strangulated bowel.

On presentation at the ER, the patient was hemodynamically stable with mild anxiety (nervousness, restlessness, difficulty concentrating). She complained of an uneasy sensation but had no pain in her abdomen. She had no history of diabetes, hypertension, or chronic illnesses. The thyroid gland function was normal. She had no history of lung infections (e.g., COVID-19), smoking, or substance abuse.

**Figure 1:**
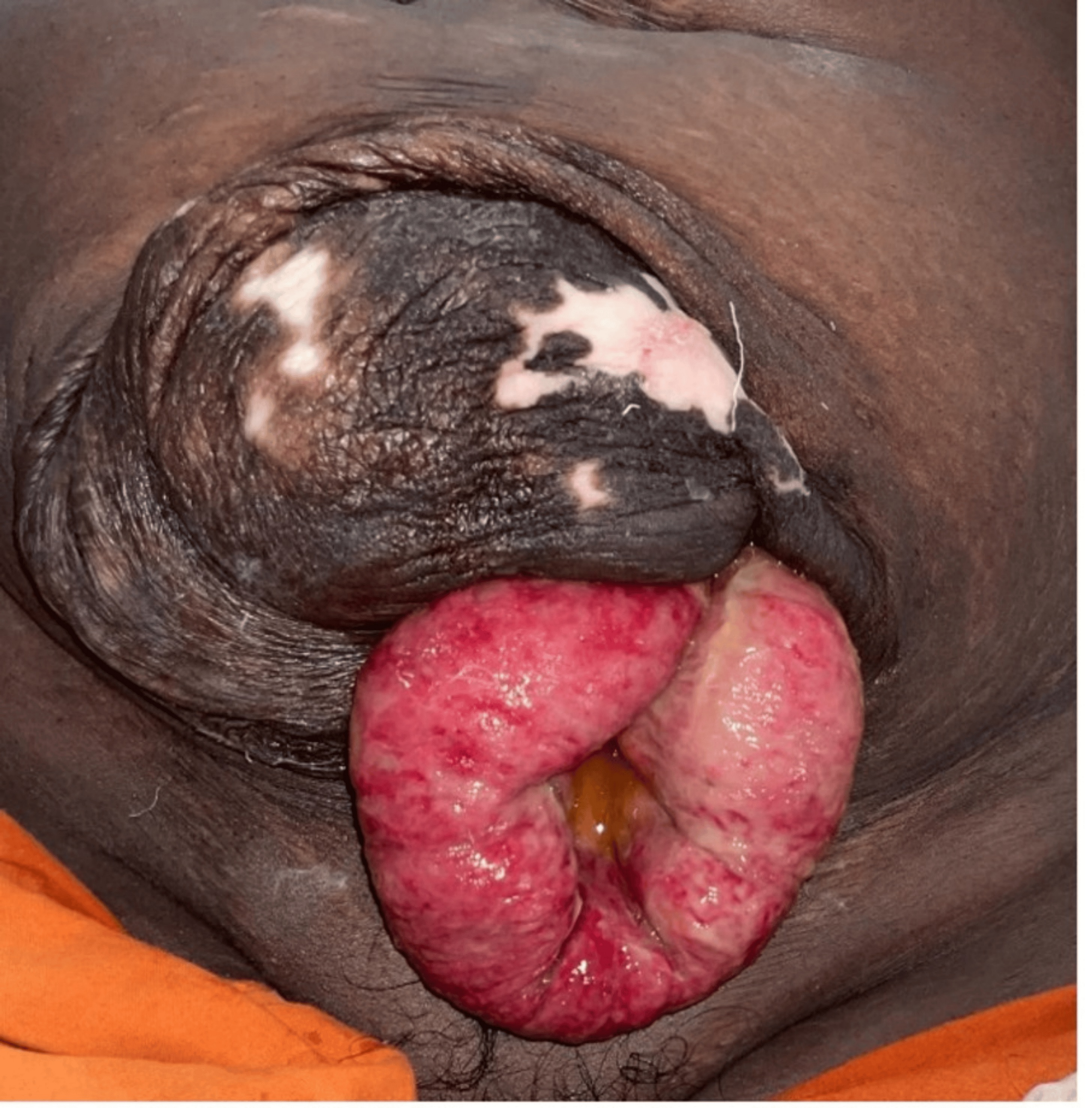
Preoperative image showing evisceration of small bowel with evidence of strangulation.

On examination, her abdomen was pendulous with a large, 20 x 20 cm swelling involving the entirety of the previous scar site (Figure 1). The overlying skin was thin, paper-like, and redundant, with patches of hyper- and hypopigmentation. There was a small, 3 x 2 cm, gangrenous defect in the skin near the inferior aspect of the swelling through which approximately 10 cm of small bowel loops were seen herniating out. The visible portion of the bowel was dusky, edematous, and nonreducible, with no visible peristalsis (Figure 1).

While waiting for laboratory parameters and after resuscitating the patient, the decision was made to incise the constricting skin at the neck of the herniating bowel loops to temporarily relieve the obstruction while the patient awaited definitive surgery. Hence, in the ER, under sterile aseptic conditions and local anesthesia, the skin defect was incised vertically by 3 cm. A clear line of demarcation could be seen between the normal and edematous bowel segments. The edematous bowel segment was dressed with paraffin gauze.

**Figure 2:**
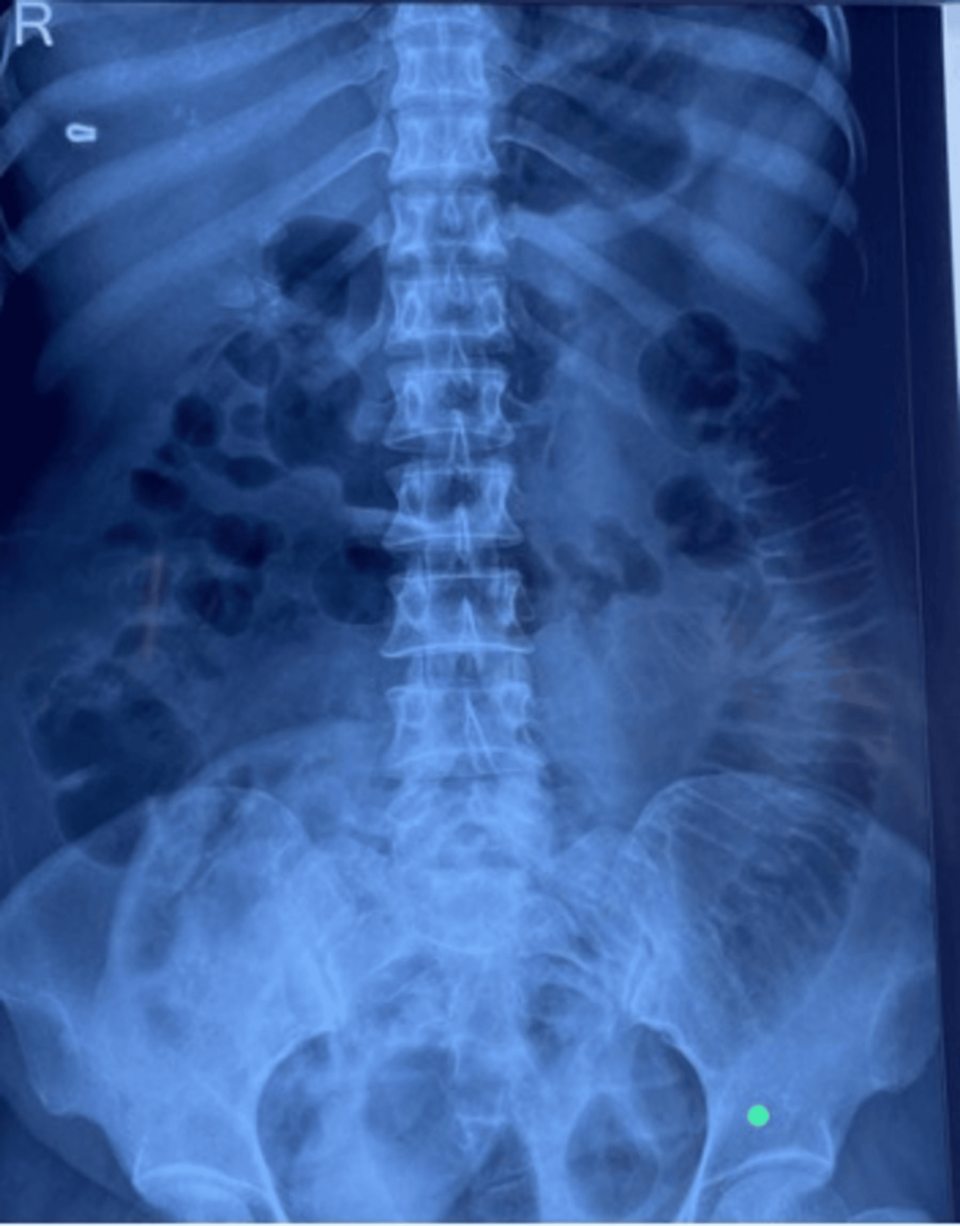
X-ray of abdomen showing dilated bowel loops.

Laboratory parameters showed leukocytosis (15000/µL). The patient’s hemogram, electrolytes, and coagulation profile were within normal limits. Arterial blood gas (ABG) analysis showed mild respiratory alkalosis, which could be attributed to the patient’s anxiety and hyperventilation. A preoperative abdominal X-ray showed grossly dilated bowel loops (Figure 2). Contrast-enhanced computed tomography (CECT) was omitted as bowel evisceration was evident, necessitating immediate surgical intervention.

The patient underwent an emergency laparotomy. The rectus sheath was severely thinned out, and the inter-recti distance at the level of the umbilicus was 12 cm. Bowel loops were traced, showing no adhesions. A 10 cm loop of jejunum, which was previously strangulated, was inspected; it was bruised but viable, and bowel viability was assessed clinically by evaluating color, peristalsis, and mesenteric pulsations. Extensive myofascial component separation was completed, and fascial edges were approximated with Prolene No. 1 suture (Johnson & Johnson MedTech, New Brunswick, New Jersey, United States). Because there was no contamination, a polypropylene mesh measuring 30 x 30 cm was placed by an onlay technique. A suction drain was placed over the fascial plane, redundant skin was excised, and an abdominoplasty (tummy tuck) was performed. 

Postoperatively, the patient recovered well and was discharged on postoperative day 5 with appropriate counseling regarding activity restrictions, wound care, signs of complications, and support for anxiety related to recovery. At the seven-day follow-up, a 3-cm margin of skin flap necrosis was observed. Wound edges were refreshed, and secondary suturing was completed. The patient had an uneventful recovery and presented with no signs of recurrence as of one year postoperatively.

## Discussion

Incisional hernias cause a significant problem for patients and surgeons as a complicating presentation for 2-20% of laparotomies [3]. The incidence of incisional hernia is highest following lower midline incisions or transverse incisions [4]. There are several factors, including surgical factors, that can pose an unusual situation [5]. This patient had multiple risk factors, including obesity and a postoperative surgical site infection with wound dehiscence resulting from a previous laparotomy.

Spontaneous rupture of an incisional hernia with bowel evisceration is a rare complication. There had only been 12 such cases reported in the literature between 2010 and 2020 [2,3,6-14]. Several cases of “spontaneous paracentesis” with bowel evisceration [15-18] have been identified in patients with tense ascites following liver cirrhosis. This condition is associated with a high mortality of approximately 40% [16]. However, no such morbidity or mortality rates are available for long-standing incisional hernia rupture.

The mechanism of bowel evisceration may be either acute or chronic. From the current case, we have observed that spontaneous evisceration of the bowel occurred following a sudden increase in intra-abdominal pressure due to lifting heavy objects, followed by a bout of coughing. In chronic, neglected cases, the overlying redundant skin may undergo progressive thinning, ulceration, or even necrosis [3]. This can ultimately lead to spontaneous rupture of the hernia sac, as observed in our patient.

An eviscerated hernia must be treated as an emergency. The patient might also suffer from a vasovagal syncope or develop symptoms of bowel obstruction and strangulation. In the current case, because the underlying bowel was viable and there was no contamination, a primary repair of the defect with onlay mesh hernioplasty was performed. The mesh was placed in an onlay position as opposed to a recto-rectus position because the rectus sheath was thinned out, making recto-rectus dissection difficult.

## Conclusions

This report presented a rare complication of incisional hernia and how it was managed successfully. The patient had a long-standing incisional hernia and presented with a spontaneous hernia rupture and bowel evisceration following a bout of coughing. Immediate resuscitation was performed, followed by an emergency laparotomy. Intraoperatively, priority was given to relieving the bowel obstruction, followed by anatomical repair. 
